# Ultrasmall superparamagnetic iron oxide nanoparticles acutely promote thrombosis and cardiac oxidative stress and DNA damage in mice

**DOI:** 10.1186/s12989-016-0132-x

**Published:** 2016-04-30

**Authors:** Abderrahim Nemmar, Sumaya Beegam, Priya Yuvaraju, Javed Yasin, Saeed Tariq, Samir Attoub, Badreldin H. Ali

**Affiliations:** 1 Departments of Physiology, College of Medicine and Health Sciences, United Arab Emirates University, P.O. Box 17666, Al Ain, United Arab Emirates; 2 Department of Internal Medicine, College of Medicine and Health Sciences, United Arab Emirates University, P.O. Box 17666, Al Ain, United Arab Emirates; 3 Department of Anatomy, College of Medicine and Health Sciences, United Arab Emirates University, P.O. Box 17666, Al Ain, United Arab Emirates; 4 Department of Pharmacology, College of Medicine and Health Sciences, United Arab Emirates University, P.O. Box 17666, Al Ain, United Arab Emirates; 5 Department of Pharmacology, College of Medicine & Health Sciences, Sultan Qaboos University, P.O. Box 35, Muscat 123, Al-Khod, Sultanate of Oman

**Keywords:** Ultrasmall superparamagnetic iron oxide nanoparticles, Thrombosis, Toxicity, Oxidative stress, Comet assay

## Abstract

**Background:**

Ultrasmall superparamagnetic iron oxide nanoparticles (USPIO) are being developed for several biomedical applications including drug delivery and imaging. However, little is known about their possible adverse effects on thrombosis and cardiac oxidative and DNA damage.

**Methods:**

Presently, we investigated the acute (1 h) effect of intravenously (i.v.) administered USPIO in mice (0.4, 2 and 10 μg/kg). Diesel exhaust particles (DEP; 400 μg/kg) were used as positive control.

**Results:**

USPIO induced a prothrombotic effect in pial arterioles and venules in vivo and increased the plasma plasminogen activator inhibitor-1 (PAI-1). Both thrombogenicity and PAI-1 concentration were increased by DEP. The direct addition of USPIO (0.008, 0.04 and 0.2 μg/ml) to untreated mouse blood dose-dependently induced in vitro platelet aggregation. USPIO caused a shortening of activated partial thromboplastin time (aPTT) and prothrombin time (PT). Similarly, DEP administration (1 μg/ml) triggered platelet aggregation in vitro in whole blood. DEP also reduced PT and aPTT. The plasma levels of creatine phosphokinase-MB isoenzyme (CK-MB), lactate dehydrogenase (LDH) and troponin-I were increased by USPIO. DEP induced a significant increase of CK-MB, LDH and troponin I levels in plasma. The cardiac levels of markers of oxidative stress including lipid peroxidation, reactive oxygen species and superoxide dismutase activity were increased by USPIO. Moreover, USPIO caused DNA damage in the heart. Likewise, DEP increased the markers of oxidative stress and induced DNA damage in the heart.

**Conclusion:**

We conclude that acute i.v. administration of USPIO caused thrombosis and cardiac oxidative stress and DNA damage. These findings provide novel insight into the pathophysiological effects of USPIO on cardiovascular homeostasis, and highlight the need for a thorough evaluation of their toxicity.

## Background

Nanotechnology is a broad interdisciplinary area that involves biochemistry, physics, biology, materials science, electrical engineering and more [1, 2]. Nanotechnology holds great promise in numerous fields, including medicine. In the latter field, it has developed rapidly to create a new application of nanotechnology in medicine known as nanomedicine which aims at the improvement of targeted drug delivery, better diagnostic and imaging techniques [1, 2]. Nanomedicine develops potential therapies designed to diagnose and treat specific diseases [1, 2]. However, concerns have been expressed about the possible adverse effects of nanomaterials [1–3].

Ultrasmall superparamagnetic iron oxide nanoparticles (USPIO, < 50 nm) have an iron oxide core that is monomer or polymer-coated/stabilized. They possess highly reactive surface, uniform particle size distribution, good suspension and the possibility of additional coating modification by conjugating with a drug [4–6]. USPIO are being developed for drug delivery and neuroimaging and imaging of tumours and metastases in the liver, spleen and bone marrow, and perfusion imaging of atherosclerotic plaque and thrombosis [4–7] However, the existing information regarding the toxicity of USPIO is rather limited, and little attention has been paid to their potential in vivo toxicity [8]. Moreover, most information on their in vivo toxicity has been derived from studies that were not specifically designed to evaluate their toxicity [7].

It is well-established that particulate air pollution and some engineered nanoparticles (such as silica) cause prothrombotic effects [9–11]. Since USPIO are being administered directly into the circulation following intravenous injection in perfusion imaging of thrombosis [6], we hypothesized that these nanoparticles can plausibly interact with the vasculature and circulating cells such as platelets and could, possibly, aggravate thrombosis. Moreover, such administered nanoparticles can also reach the heart and cause oxidative stress and DNA damage. Therefore, the aim of this study was to investigate the acute (1 h) effect of intravenously (i.v.) administered USPIO in mice (0.4, 2 and 10 μg/kg) on thrombosis in vivo and in vitro and cardiac oxidative stress and DNA damage. Appropriate positive control particles were used in the present study, i.e. diesel exhaust particles (DEP) which are known to cause thrombosis, cardiotoxicity and oxidative stress [12–14]. The dose of DEP tested (400 μg/kg or 0.4 mg/kg) is comparable to that used recently by Tabor et al. for i.v. injection (0.5 mg/kg) in rats [12].

## Methods

### USPIO and DEP

As described by the manufacturer (Sigma, St. Louis, MO, USA), the USPIO (magnetite, Fe_3_O_4_) used in the present study have a particle size ranged between 4 and 6 nm (diameter 5 nm, by transmission electron microscopy), density 5 mg/mL in H_2_O [includes total weight of nanoparticles plus <1.0 % modified short chain polyethylene glycol (PEG) stabilizing ligands], 1 mg/ml Fe and magnetization >25 emu/g, at 4500Oe.

To confirm their size, we performed transmission electron microscopy analysis of the USPIO. Thus, droplets (10 μL) of a suspension of 0.1 mg of USPIO in 500 μL were placed on matured formvar/carbon film for 30 s. The samples were then drained and inverted onto droplets of ultrapure water for 1 h before being drained, dried, and examined in a Philips CM10 Transmission Electron Microscope.

The DEP were obtained from the National Institute of Standards and Technology (NIST, Gaithersburg, MD, USA), and were suspended in sterile saline (NaCl 0.9 %). These particles were previously analysed by transmission electron microscopy, and shown to have a substantial amount of ultrafine (nano) sized particle aggregates, and larger particle aggregates [15].

The endotoxin concentration in the USPIO solution, DEP and saline used was quantified, as described by the manufacturer, by chromogenic Limulus Amebocyte Lysate (Pierce, Rockford, IL) test. The concentrations were lower than the detection limit (0.1 EU/ml) in the saline, and USPIO and DEP solutions.

### Animals and i.v. administration of USPIO or DEP

This project was reviewed and approved by our Institutional Review Board and experiments were performed in accordance with protocols approved by the Institutional Animal Care and Research Advisory Committee.

USPIO or DEP were suspended in normal saline (NaCl 0.9 %). To minimize aggregation, particle suspensions were always sonicated (Clifton Ultrasonic Bath, Clifton, New Jersey, USA) for 15 min and vortexed before their dilution and prior to i.v. administration.

BALB/C mice (Taconic Farms Inc., Germantown, NY, USA) weighing 20–25 g were housed in light (12-h light:12-h dark cycle) and temperature-controlled (22 ± 1 °C) rooms. They had access to commercial laboratory chow and tap water *ad libitum*.

The mice were anesthetized with urethane (1 mg/g body weight, i.p.), and 2 F catheter (Portex, Hythe, UK) was inserted in the right jugular vein. Either USPIO suspensions (0.4, 2 and 10 μg/kg) or DEP (400 μg/kg) or saline-only were i.v. injected (100 μl), and 1 h later various cardiovascular parameters were assessed.

### Experimental thrombosis model in pial arterioles and venules

The assessment of thrombosis has been performed according to a previously described technique [16–19]. Briefly, the trachea was intubated after induction of anaesthesia with urethane (1 mg/g body weight, i.p.), and a 2 F venous catheter (Portex, Hythe, UK) was inserted in the right jugular vein for the administration of fluorescein (Sigma, St. Louis, MO, USA). After that, a craniotomy was first performed on the left side, using a microdrill, and the dura was stripped open. Only untraumatised preparations were used, and those showing trauma to either microvessels or underlying brain tissue were discarded. The animals were then placed on the stage of a fluorescence microscope (Olympus, Melville, NY, USA) attached to a camera and DVD recorder. A heating mat was placed under the mice and body temperature was raised to 37 °C, as monitored by a rectal thermoprobe connected to a temperature reader (Physitemp Instruments, NJ, USA). The cranial preparation was moistened continuously with artificial cerebrospinal fluid of the following composition (mM): NaCl 124, KCl 5, NaH_2_PO_4_ 3, CaCl_2_ 2.5, MgSO_4_.4, NaHCO_3_ 23 and glucose 10, pH 7.3–7.4. A field containing arterioles and venules 15–20 μm in diameter was chosen. Such a field was taped prior to and during the photochemical insult, which was carried out by injecting fluorescein (0.1 ml/mouse of 5 % solution) via the jugular vein, which was allowed to circulate for 30–40 s. The cranial preparation was then exposed to stabilized mercury light. The combination produces endothelium injury of the arterioles and venules. This, in turn, causes platelets to adhere at the site of endothelial damage and then aggregate. Platelets aggregate and the thrombus forms and grows in size until complete vascular occlusion. The time, in seconds, was measured from the photochemical injury until full vascular occlusion (time to flow stop) in venules. One hour before the administration of fluorescein, 100 μl of saline (control) or saline containing either USPIO or DEP was administered via the jugular vein. The animals were euthanized at the end of the recording by an overdose of anaesthesia.

### Blood collection and analysis

In separate experiments, new mice were anesthetized and were given i.v. saline (control) or saline containing either USPIO or DEP as described above. One h later, the animals’ blood was drawn from the inferior vena cava in EDTA (4 %) and centrifuged at 4 °C for 15 min at 3000 rpm, and the plasma samples obtained were stored at−80 °C until further analysis.

### Determination of systemic markers of fibrinolysis and myocardial membrane damage

The concentration of plasminogen activator inhibitor-1 (PAI-1) (Molecular Innovation, Southfield, MI, USA) was determined using ELISA Kit. Creatine phosphokinase-MB isoenzyme (CK-MB), troponin-I and lactate dehydrogenase (LDH) were measured spectrophotometrically using kits (Roche, Basel, Switzerland).

### Measurement of cardiac markers of oxidative stress

The hearts obtained from the animals used above were quickly rinsed with ice-cold PBS (pH 7.4) before homogenization in 0.1 M phosphate buffer pH 7.4 containing 0.15 M KCl, 0.1 mM EDTA, 1 mM DTT and 0.1 mM phenylmethylsulfonylfluoride at 4 °C. The homogenates were centrifuged for 20 min at 14,000 rpm to remove cellular debris and supernatants were used for further analysis. Protein content was measured by Bradford’s method as described before [20–22].

Measurement of reactive oxygen species (ROS): ROS were measured in the whole cardiac tissue homogenates which were obtained as described above using 2’, 7’- Dichlorofluorescein diacetate (DCFDA; Molecular Probes, Eugene, OR, USA) as a fluorescent probe as described before [20–22]. The results were normalized as ROS produced per mg of protein.

NADPH-dependent membrane LPO in the heart homogenate was determined using TBARS kit purchased from Cayman Chemical Company (Ann Arbor, MI, USA). Measurement of superoxide dismutase (SOD) was performed by an ELISA technique with commercially available kit (Cayman Chemicals, Michigan, USA).

### In vitro platelet aggregation in mouse whole blood

The platelet aggregation assay in whole blood was performed, with slight modification, as described before [23]. After anaesthesia, blood from untreated mice was withdrawn from the inferior vena cava and placed in citrate (3.2 %), and 100-μl aliquots were added to the well of a Merlin coagulometer (MC 1 VET; Merlin, Lemgo, Germany). The blood samples were incubated at 37.2 °C with either saline (control) or USPIO (0.008, 0.04 and 0.2 μg/ml) or DEP (1 μg/ml) for 3 min, and then stirred for another 3 min. At the end of this period, 25-μl samples were removed and fixed on ice in 225 ml cellFix (Becton Dickinson). After fixation, single platelets were counted in a VET ABX Micros with a mouse card (ABX, Montpellier, France). The degree of platelet aggregation following USPIO or DEP exposure was expressed as a ℅ of control (saline-treated blood).

### In vitro measurement of prothrombin time (PT) and activated partial thromboplastin time (aPTT) in plasma

One hour following either saline or USPIO or DEP administration, blood was withdrawn from each mouse, as described above. The PT was measured [24–26] on freshly collected, platelet-poor plasma with human, relipidated, recombinant thromboplastin (Recombiplastin; Instrumentation Laboratory, Orangeburg, NY, USA) in combination with a Merlin coagulometer MC 1 VET (Merlin, Lemgo, Germany). The aPTT was measured [24–26] with automated aPTT reagent (BioMerieux, Durham, NC, USA) using a Merlin coagulometer MC 1 VET (Merlin, Lemgo, Germany). Normal plasma used as reference for both the PT and aPTT was prepared by pooling equal portions of platelet-poor plasmas from the blood of six untreated mice.

### DNA damage assessment in the heart by COMET assay

Immediately after sacrifice, the heart was removed from each animal. Single-cell suspensions of the different hearts were obtained according to the method described by de Souza et al.[27]. Each collected organ was washed in a chilled medium (RPMI 1640, 15 % DMSO, 1.8 % (w/v) NaCl). The heart tissues were placed in 1.5 ml medium and chopped finely into pieces in a Petri dish using scissors. The pieces were allowed to settle and the supernatant was collected in a 15 ml tube. The obtained cell suspension was centrifuged at 1000 rpm for 5 min at 4^0^ C. The supernatant was discarded and the pellets were resuspended in 0.5 ml of the medium. The cell suspensions were mixed with low melting point agarose solution (0.65 %) and spread onto agarose (1.5 %)–precoated microscope slides. For each treatment five slides were prepared, which were incubated in ice cold lysis buffer (2.5 M NaCl, 10 mM Tris, 100 mM EDTA, 1 % Triton X-100 and 10 % DMSO) at 4^0^ C for at least 1 h to remove the cell membranes. After the incubation, slides were placed in a horizontal electrophoresis unit and incubated in electrophoresis buffer (0.2 M EDTA, 5 M NaCl, pH 10) for 20 min for DNA unwinding and the expression of alkali labile sites. Then, electrophoresis was conducted for 20 min at 25 V and 300 mA. After that, the slides were neutralized with Tris buffer (0.4 M Trizma base, pH 7.5) for 5 min and washed with methanol. Then the slides were stained with propidium iodide, as previously described [28, 29]. All these steps were performed in darkness to prevent additional DNA damage. The slides were mounted on a fluorescent microscope and cell scoring was performed. Fifty cells from each treatment were scored and analyzed for DNA migration and the average of the five slides from each group was calculated. The measurement of length of the DNA migration (i.e. diameter of the nucleus plus migrated DNA) was calculated using image analysis Axiovision 3.1 software (Carl Zeiss, Canada) [29, 30].

### Statistics

All statistical analyses were performed with GraphPad Prism Software version 5 (San Diego, CA, USA). To determine whether parameters were normally distributed, the Kolmogorov–Smirnov statistic normality test was applied. Comparisons between groups were performed by one way analysis of variance (ANOVA), followed by Newman-Keuls multiple-range tests. Data which were not normally distributed were analyzed with Kruskal-Wallis test followed by Dunn’s multiple comparison test. *P*-values <0.05 are considered as significant.

## Results

### Transmission electron microscopy analysis of USPIO

**Fig. 1:**
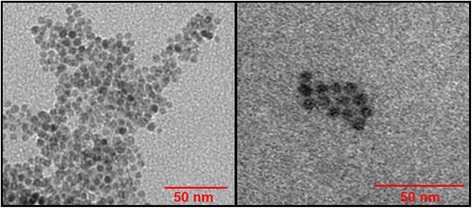
Transmission electron micrographs of the ultrasmall superparamagnetic iron oxide nanoparticles suspension showing the presence of nanosized particles

Transmission electron microscopy of the USPIO revealed a homogeneous particle size of 5 nm (range between 4 and 6 nm) (Fig. 1). This confirms the size provided by the manufacturer.

### Effect of USPIO or DEP on photochemically-induced thrombosis in pial arterioles and venules

**Fig. 2:**
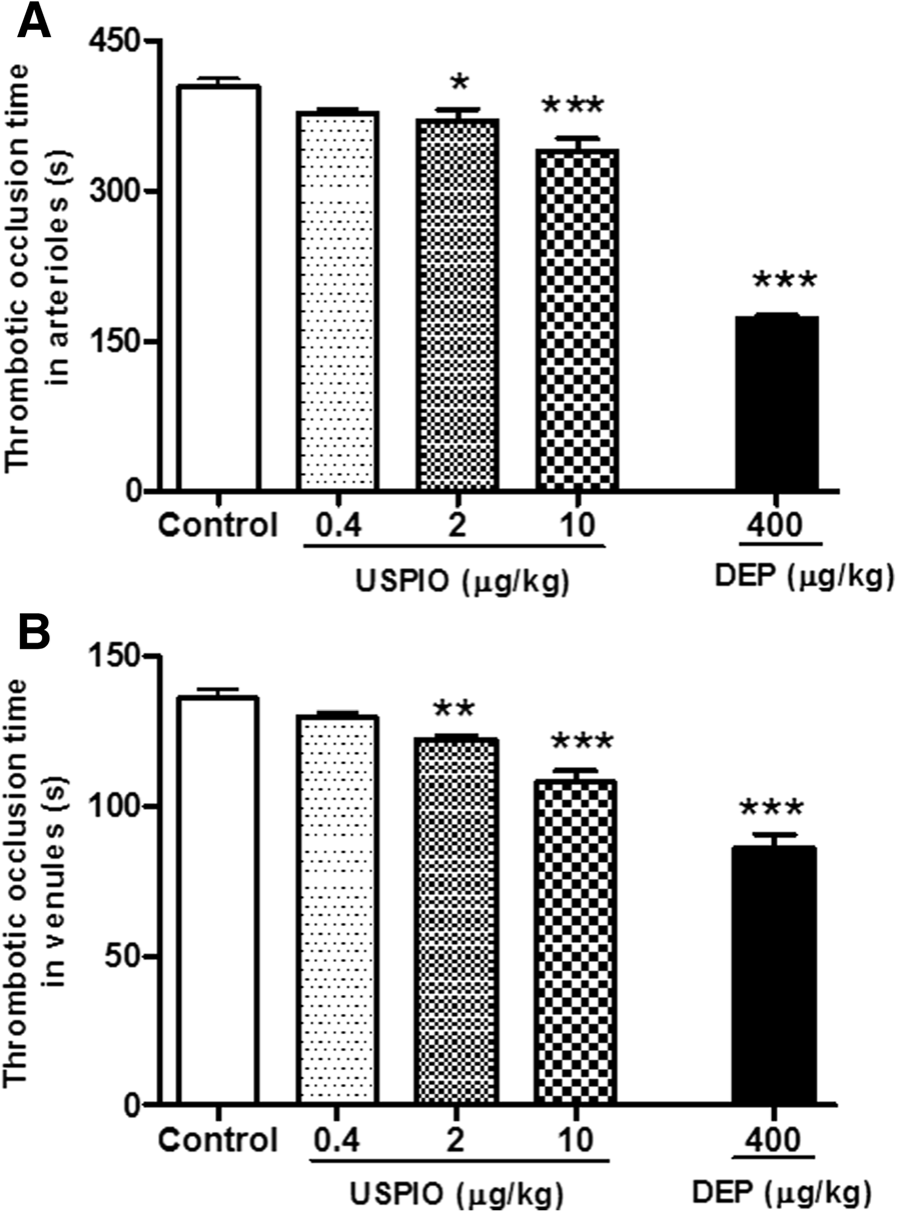
Thrombotic occlusion time in pial arterioles (**a**) or venules (**b**) 1 h after the intravenous administration of ultrasmall superparamagnetic iron oxide nanoparticles (USPIO), diesel exhaust particles (DEP) or saline (control) in mice. *P < 0.05, **P < 0.01 and ***P < 0.001 compared with the corresponding saline-treated group. Data are mean ± SEM (*n* = 6–8)

Figure 2a illustrates that the i.v. administration of USPIO caused a significant shortening of the thrombotic occlusion time in the pial arterioles at the dose of 2 μg/kg (*P* < 0.05) and 10 μg/kg (*P* < 0.001). Likewise, Fig. 2b shows that the i.v. injection of USPIO induced a significant shortening of the thrombotic occlusion time in pial venules at the dose of 2 μg/kg (*P* < 0.01) and 10 μg/kg (*P* < 0.001). At the dose studied (400 μg/kg), DEP induced more shortening in the thrombotic occlusion time in pial arterioles (*P* < 0.001) and venules (*P* < 0.001).

### Effect of USPIO or DEP on PAI-1 concentration in plasma

**Fig. 3:**
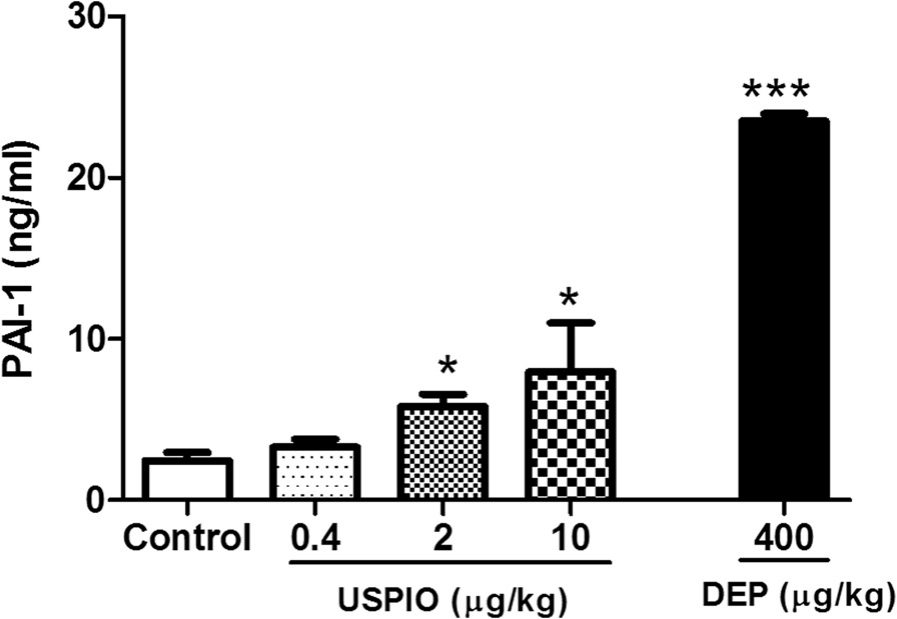
Plasminogen activator inhibitor-1 (PAI-1) concentrations in plasma, 1 h after the intravenous administration of ultrasmall superparamagnetic iron oxide nanoparticles (USPIO), diesel exhaust particles (DEP) or saline (control) in mice. *P < 0.05 and ***P < 0.001 compared with the corresponding saline-treated group. Data are mean ± SEM (*n* = 6)

The plasma concentration of PAI-1, an endogenous factor of fibrinolysis, was significantly increased following USPIO administration of 2 μg/kg (*P* < 0.05) and 10 μg/kg (*P* < 0.05) compared with the control group (Fig. 3). DEP induced a significant increase in PAI-1 concentration (*P* < 0.001).

### In vitro effect of USPIO or DEP on platelet aggregation in whole blood

**Fig. 4:**
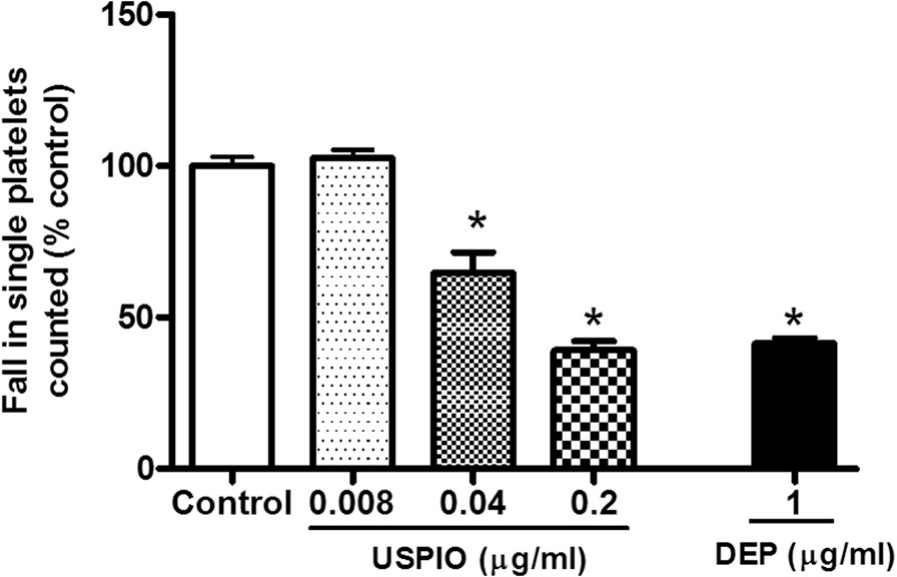
Direct in vitro effect after the administration of ultrasmall superparamagnetic iron oxide nanoparticles (USPIO), diesel exhaust particles (DEP) or saline (control) on platelet aggregation in whole blood of untreated mice. Platelet aggregation in untreated whole blood 3 min after the addition of either saline or USPIO was assessed. The degree of platelet aggregation following USPIO exposure was expressed as a percent of control (saline-treated blood). Data are mean ± SEM (*n* = 4). *P < 0.001 compared with saline-treated blood within the same group

Figure 4 illustrates the effect of various concentrations of USPIO (0.008, 0.04 and 0.2 μg/ml blood) on platelet aggregation in whole blood in vitro. A concentration-dependent and significant platelet aggregation was observed after the addition of USPIO to whole blood at concentrations of 0.04 μg/ml (*P* < 0.001) and 0.2 μg/ml (*P* < 0.001). Likewise, DEP administration (1 μg/ml) in vitro caused a significant platelet proaggregatory effect in whole blood (*P* < 0.001).

### Effect of USPIO or DEP on PT and aPTT

**Fig. 5:**
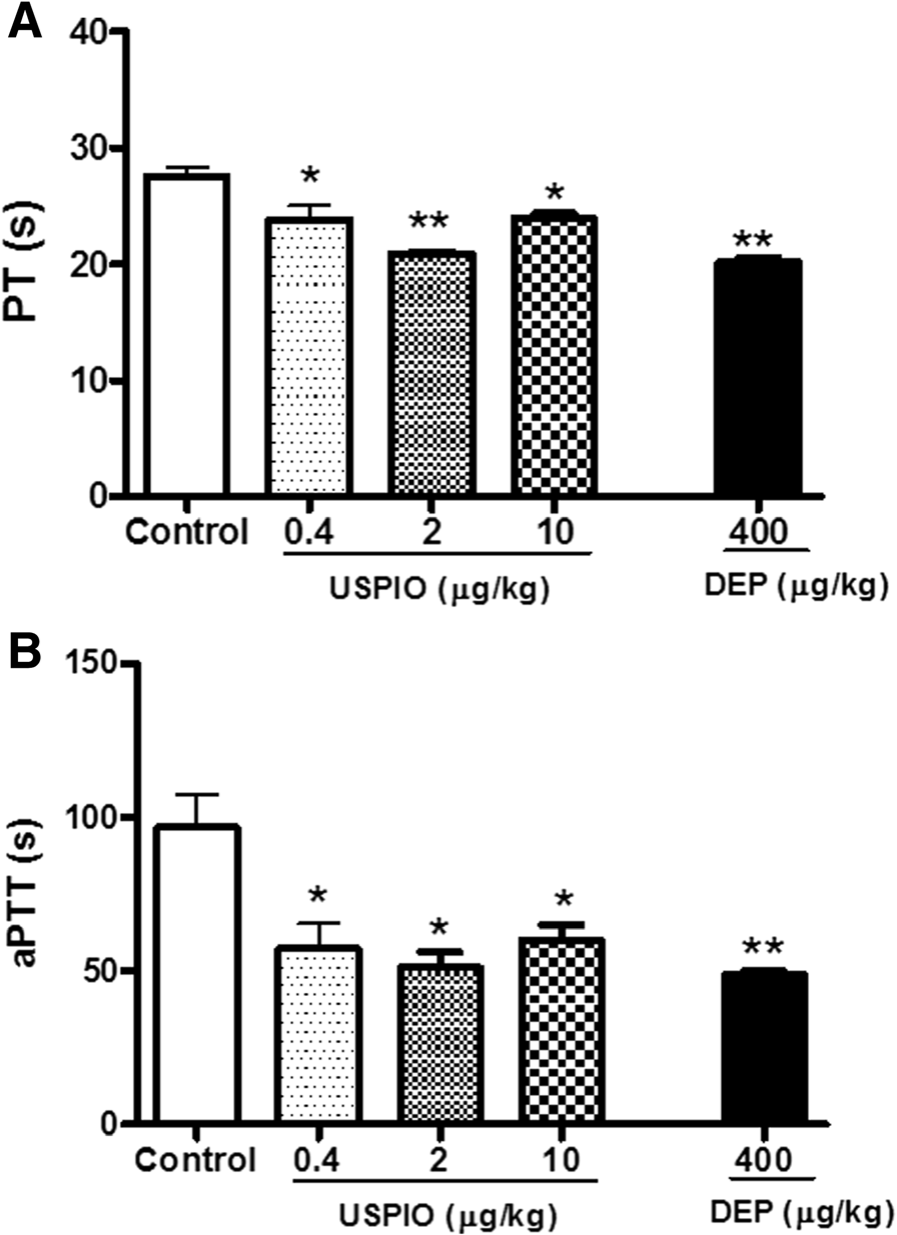
Prothrombin time (PT, **a**) and activated partial thromboplastin time (aPTT, **b**) measured 1 h after the intravenous administration of ultrasmall superparamagnetic iron oxide nanoparticles (USPIO), diesel exhaust particles (DEP) or saline (control) in mice. *P < 0.01 and **P < 0.001 compared with the corresponding saline-treated group. Data are mean ± SEM (*n* = 4–6)

The PT and aPTT measured in plasma obtained from mice i.v. administered with USPIO or DEP are shown in Fig. 5.

In comparison with the value obtained for the control group, the i.v. administration of USPIO caused a significant shortening of PT. The level of significance was achieved at 0.4 μg/kg (*P* < 0.01), 2 μg/ml (*P* < 0.001) and 10 μg/ml (*P* < 0.01) (Fig. 5a). Similarly, the administration of USPIO induced a significant shortening of aPTT at 0.4 μg/ml (*P* < 0.01), 2 μg/ml (*P* < 0.01) and 10 μg/ml (*P* < 0.01) (Fig. 5b). DEP significantly reduced the PT (*P* < 0.001) and aPTT (*P* < 0.001).

### Effect of USPIO or DEP on plasma activities of LDH, CK-MB and troponin-I

**Fig. 6:**
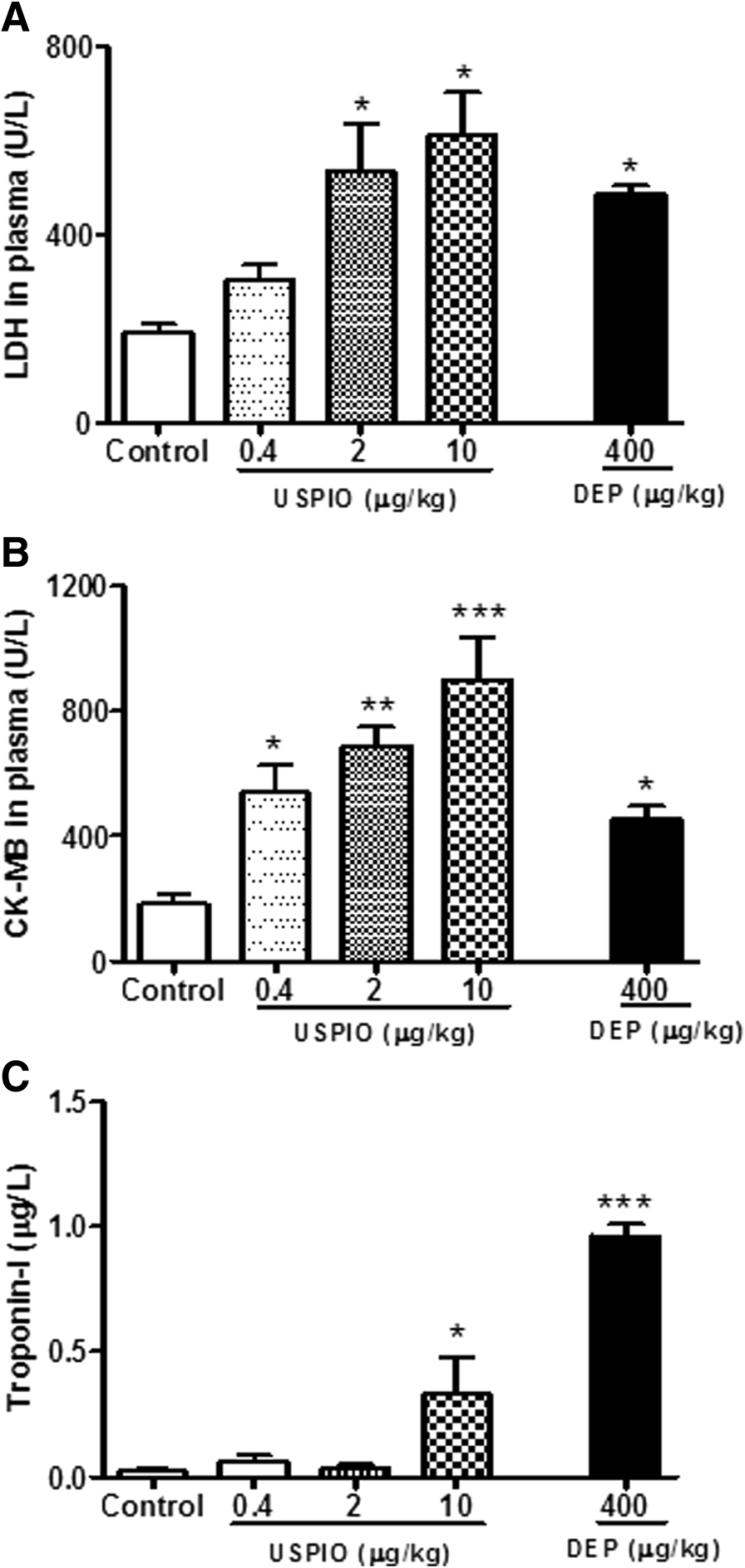
Lactate dehydrogenase (LDH, **a**) and creatine phosphokinase-MB (**b**) and troponin-I (**c**) in plasma 1 h after the intravenous administration of ultrasmall superparamagnetic iron oxide nanoparticles (USPIO), diesel exhaust particles (DEP) or saline (control) in mice. *P < 0.05, **P < 0.01 and ***P < 0.001 compared with the corresponding saline-treated group. Data are mean ± SEM (*n* = 4–6)

The i.v. administration of USPIO caused a significant and dose-dependent increase in the levels of LDH, CK-MB and troponin-I in plasma (Fig. 6a–c). For the LDH, the increase was statistically insignificant at 0.4 μg/ml, but it was significant at 2 μg/ml (*P* < 0.05) and 10 μg/ml (*P* < 0.05) (Fig. 6a). Regarding CK-MB, the level of significance was achieved at all the studied doses, i.e. 0.4 μg/ml (*P* < 0.05), 2 μg/ml (*P* < 0.01) and 10 μg/ml (*P* < 0.001) (Fig. 6b). The troponin-I levels were increased at 10 μg/ml (*P* < 0.05) (Fig. 6c). Similarly, DEP significantly augmented the levels of LDH (*P* < 0.05), CK-MB (*P* < 0.05) and troponin I (*P* < 0.001).

### Effect of USPIO or DEP on cardiac oxidative stress

**Fig. 7:**
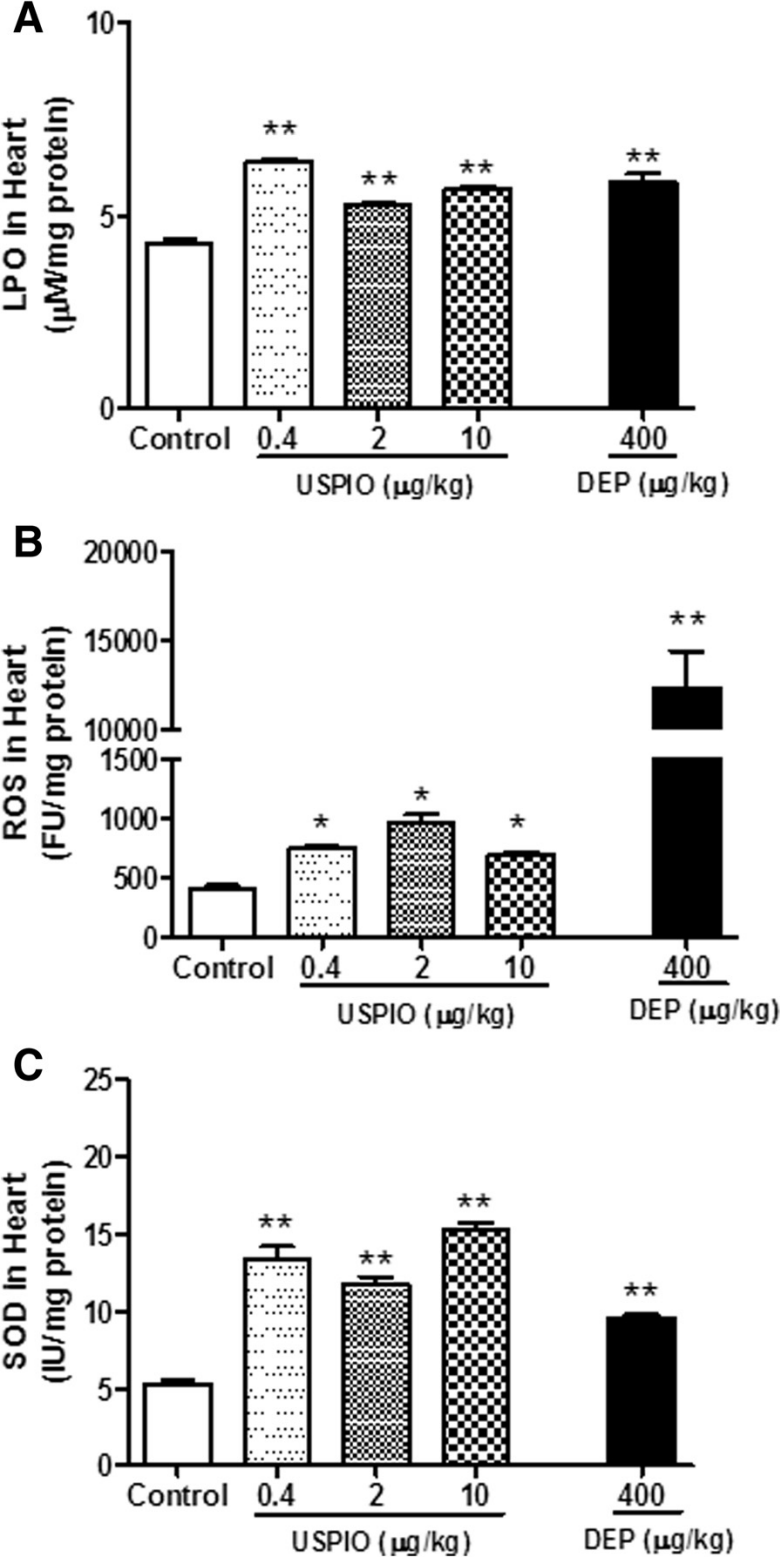
Lipid peroxidation (LPO, **a**), reactive oxygen species (ROS, **b**) and superoxide dismutase (D) levels in heart tissues 1 h after the intravenous administration of ultrasmall superparamagnetic iron oxide nanoparticles (USPIO), diesel exhaust particles (DEP) or saline (control) in mice. *P < 0.01 and **P < 0.001 compared with the corresponding saline-treated group. Data are mean ± SEM (*n* = 6–8)

Figure 7a illustrates that the i.v. administration of USPIO induced a significant increase of lipid peroxidation in heart tissue at 0.4 μg/ml (*P* < 0.001), 2 μg/ml (*P* < 0.001) and 10 μg/ml (*P* < 0.001) compared with control group. ROS levels in heart tissue were increased at all the studied doses of USPIO (*P* < 0.01) (Fig. 7b). Along with the increase of LPO and ROS, the levels of the antioxidant SOD were significantly increased (*P* < 0.001) at all the studied doses (Fig. 7c). DEP induced a significant increase of all the assessed markers of oxidative stress, i.e. LPO (*P* < 0.001), ROS (*P* < 0.001) and SOD (*P* < 0.001).

### Effect of USPIO on cardiac DNA damage

**Fig. 8:**
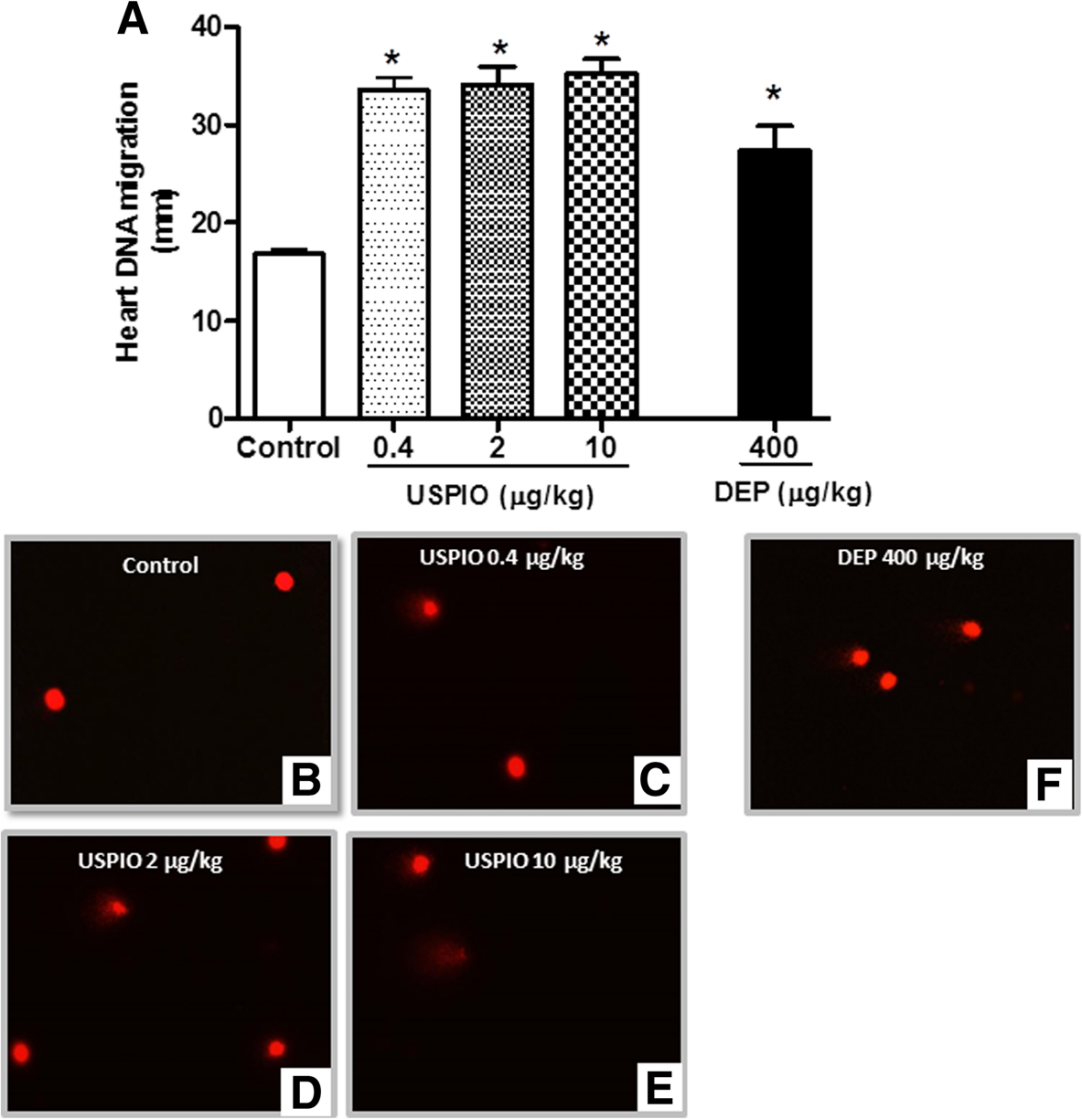
DNA migration in the heart tissues, 1 h after the intravenous administration of ultrasmall superparamagnetic iron oxide nanoparticles (USPIO), diesel exhaust particles (DEP) or saline (control) in mice. Images illustrating the quantification of the DNA migration by the Comet assay under alkaline conditions, in control (**b**), USPIO (**c**–**e**) or DEP (**f**). *P < 0.001 compared with the corresponding saline-treated group. Data are mean ± SEM (*n* = 5)

Figure 8 shows that compared with control group, the i.v. injection of USPIO induced DNA damage in the heart at 0.4 μg/ml (*P* < 0.001), 2 μg/ml (*P* < 0.001) and 10 μg/ml (*P* < 0.0.01). Likewise, DEP caused significant DNA damage in the heart (*P* < 0.001).

## Discussion

We showed here that acute (1 h) i.v. administration of USPIO caused prothrombotic events in pial arterioles and venules in vivo, platelet aggregation in whole blood in vitro and shortening of PT and aPTT. The concentration of PAI-1 was increased by USPIO. Markers of myocardial membrane damage in plasma including CK-MB, LDH and troponin-I were significantly increased by USPIO. Moreover, USPIO induced oxidative stress in the heart evidenced by increase of LPO, ROS and SOD, and caused cardiac DNA damage. All the aforementioned endpoints were increased by the administration of the positive control particles, namely DEP.

Commercially available USPIO are surface coated with materials such as silicon, dextran, citrate and PEGylated starch and are mainly used as contrast agents in target organs [8]. In the present study, we tested 5 nm USPIO with PEG stabilizing ligands. PEG has been broadly utilised in the formulation of nanoparticles for biomedical applications, both because of its biocompatibility and its effectiveness in masking nanoparticles from opsonins [31]. These nanoparticles were administered i.v. to the mice. This route of exposure imitates their potential use in clinical settings [4]. Moreover, their small size allow them to cross the capillary beds and gain access to various organs such as lung, brain kidney, spleen, liver and heart [32]. Previously, when dextran-coated superparamagnetic iron oxide nanoparticles were examined as a contrast agent in subjects, their adverse effects (headache, back pain, vasodilatation, and urticarial) were reported to be mild or moderate in severity and of short duration [33]. A dose of 2.6 mg of iron per kg was applied [33]. In experimental arthritis model in mice, doses of 3.3 mg Fe/kg or 33.3 mg Fe/kg were injected i.v. [34]. The doses of USPIO (0.4, 2 and 10 μg per kg) used in our study in mice have low iron content of 0.0004, 0.002 and 0.01 μg Fe per kg, respectively. However, the amount of iron injected is not the sole parameter that determines the toxicity of nanoparticles in general [3, 35]. In fact, it is well established that nanoparticles have unique features including their very small size, large surface area and cellular penetration capacity which are responsible for their specific biological responses that differ from larger particle of similar chemical composition [3, 35, 36]. Recently, it has been shown that i.v. injected polyacrylic acid coated γ-Fe_2_O_3_ nanoparticles (10 mg/kg) in healthy BALB/cJ mice induced a decrease in the mean arterial blood pressure (MAP) associated with a decreased contractility of small mesenteric arteries [37]. In anaesthetized pigs, it has been demonstrated that dimercaptosuccinic acid coated superparamagnetic iron oxide nanoparticles (MF66-labelled 12 nm, core nominal diameter and OD15 15 nm); at 0.5, or 2.0 mg/kg injected i.v. induced a transient yet significant decrease in MAP [38]. Particles remained in the circulation for up to 30 min and were accumulated in the liver, spleen and lungs [38]. With regard to human exposure situation, pharmacokinetic data related to the liposome-encapsulated doxorubicin nanoparticles, used in cancer therapy, reported plasma concentrations of more than 10 μg/ml [39]. The last concentration is much higher than that used in the present in vivo study. In fact, the highest dose of nanoparticles used here which is 10 μg/kg (0.25 μg/mouse of 25 g) would correspond to approximately 0.16 μg/ml, assuming a circulating mouse blood volume of 1.5 ml. One of the reasons why we wanted to use lower dose of USPIO in our study is because we wanted to test the possible exacerbating effect of USPIO on an in vivo photochemical injury model of thrombosis in the pial cerebral arterioles and venules. In clinical settings, patients who will be potentially injected with these nanoparticles, for diagnosis or treatment purposes, will likely have pre-existing diseases (e.g. cardiovascular diseases). Therefore, we can speculate that those patients may conceivably exhibit increased susceptibility to adverse cardiovascular effect of USPIO as compared with healthy peoples. In this sense, the minor photochemical trauma to endothelium used in our experiments may have pathophysiological relevance [40]. Superficial erosion, or microscopic areas of desquamation of endothelial cells, occurs frequently in both humans and in animals with experimentally-induced atherosclerosis [40]. The doses of DEP used in vivo (400 μg/kg or 0.4 mg/kg) and in vitro (1 μg/ml) are similar to those used recently to study the prothrombotic effects of DEP in vivo and in vitro [12, 13]. Our data show that the i.v. administration of USPIO for 1 h caused a significant and dose-dependent shortening of the thrombotic occlusion time, showing that USPIO have prothrombotic effect. Several previous studies have reported the use of USPIO for perfusion imaging of atherosclerotic plaque and thrombosis [7]. For example, in a recent study, USPIO were injected i.v. at different time points after photothrombotic infarction in mice, and it was possible to follow vessel occlusion in vivo by USPIO-enhanced high-field MRI [41]. It has been reported that i.v. administration of polystyrene nanoparticles in hamsters, rats and mice, and DEP in rats and mice cause in vivo thrombogenicity [15, 36, 42–44]. Nevertheless, the possible prothrombotic effect of USPIO has, as far as we are aware, never been reported so far.

In the present study, we also found a significant increase of circulating PAI-1 following the administration of USPIO. PAI-1 is the most potent endogenous inhibitor of fibrinolysis and is involved in the pathogenesis of several cardiovascular diseases [45, 46]. An increase of PAI-1 has been observed following exposure to either DEP [47] or silica nanoparticles [11].

Our model of thrombosis depends on platelet activation and aggregation [17]. Consequently, we wanted to verify whether and to what extent can USPIO directly activate platelet and cause their aggregation in whole blood in vitro. Since the highest dose of nanoparticles tested, presently, in vivo (10 μg/kg or 0.25 μg/mouse) would correspond to approximately 0.16 μg/ml blood, we selected to test in vitro concentrations which are relevant to our in vivo study, i.e. 0.2 μg/ml being the highest concentration and 2 lower ones (0.04 and 0.008 μg/ml). Our data show that low concentrations of USPIO cause a significant and dose-dependent proaggregatory effect, confirming the in vivo prothrombotic findings. Although USPIO are developed for i.v. administration, and hence, the study of their interaction with circulating cells such as platelets is critical, little is known about their potential effect on platelet aggregation. Several studies focused on designing functionalized USPIO to target activated platelets for the study atheroma pathogenesis [7, 48]. However, other types of particles such silica, polystyrene, DEP or TiO2 have previously been reported to induce platelet aggregation in vitro [11, 23, 36, 44, 49, 50]. Furthermore, we demonstrated the activation of PT and aPTT in plasma of mice in vivo administered with various doses of USPIO. This effect reflects hypercoagulability caused by USPIO and confirm the in vivo and in vitro prothrombotic effects of these nanoparticles. High concentration of maghemite nanoparticles bioferofluids (0.38 mg/ml Fe_2_O_3_) has been reported to shorten PT but, on the other hand, it caused prolongation of aPTT [51]. Here, we also found that DEP caused a shortening of PT and aPTT, confirming our earlier findings [13]. Our data showed significant increments in cardiac enzyme levels of LDH, CK-MB and troponin-I (at 10 μg/kg), which may be attributed to the myocardial membrane damage produced by USPIO and the leakage of these enzymes into blood [52]. To gain more insight into the cardiac toxicity of USPIO, we assessed the effect of these nanoparticles on oxidative stress and DNA damage in the heart tissue. Oxidative stress is a key pathological process in a variety of pathophysiological conditions affecting various organs such as the lung, heart, kidney or liver and is characterized by the formation of a wide range of ROS, which can cause severe DNA, protein, and lipid damage leading to cellular dysfunction and death [53]. Iron oxide nanoparticles found either in the environment or in biomedical settings are readily taken up by the cells and cause the generation of free radicals leading to oxidative stress [8, 54]. It has been shown that even stabilized iron oxide particles with coatings such as dextran or citric acid may cause oxidative stress [55]. More recently, to mitigate oxidative stress-induced by iron oxide nanoparticles, antioxidant polymer poly(trolox) was formulated into nanoparticles coated with an antibody directed towards platelet endothelial cell adhesion molecule-1 [56]. These active targeting nanoparticles have shown to adhere to human umbilical vein endothelial cells, internalize, and reduce oxidative stress induced by iron oxide particles [56]. Nevertheless, the effect of USPIO on oxidative stress and DNA damage in the heart has never been reported so far. Our data show a significant increase in LPO and ROS in the heart. Moreover, we found a significant increase of the antioxidant SOD in the heart. This indicates that the development of oxidative stress is accompanied by an adaptive response that counterbalances the potentially damaging activity of oxygen free radicals by antioxidant defense mechanisms. Increase of SOD following exposure to silica nanoparticles has been recently reported in mouse erythrocytes [57] and in the heart of mice exposed to cigarette smoke [21]. Likewise, in the present study, DEP induced a significant increase of all the markers of oxidative stress studied. For evaluating genetic damage, the gel electrophoresis of a single cell (Comet assay) was used to assess DNA damage in the heart following USPIO administration. Our data show a significant DNA damage in the heart after the acute administration of USPIO. DEP caused similar effect. We speculate that ROS generation and oxidative stress caused by USPIO induced DNA damage in the heart. Such an effect has not been reported before. An in vitro study showed that iron oxide nanoparticles caused DNA damage and apoptosis through ROS generation in human breast cancer cell line [58]. A recent study in rats using high doses of Fe_2_O_3_-30 nm nanoparticles (500–2000 mg/kg) given orally did not assess DNA damage in heart but showed no genotoxicity in circulating leukocytes between 6 h and 72 h post-exposure [59].

## Conclusion

We conclude that acute i.v. administration of USPIO exerts procoagulatory effect in vivo and in vitro and cause cardiac oxidative stress and DNA damage. These findings provide novel insight into the pathophysiological effects of USPIO on cardiovascular system, and highlight the need for a comprehensive assessment of their toxicity before their clinical use. Additional studies are needed to investigate the effect of various coating such as dextran on the toxicity of USPIO and the mechanisms underlying the effects of USPIO on platelet activation in human blood.
